# Parental perception of nocturnal enuresis in a local region of Saudi Arabia

**DOI:** 10.25122/jml-2023-0423

**Published:** 2024-01

**Authors:** Haytham Mohammed Alarfaj, Abdullah Almaqhawi, Ahmed Hassan Kamal, Mohammed Saad Bu Bshait, Ahmad Al Abdulqader, Mohammed Albarqi, Mohammed Almoghnam, Zahra Ali Alhaddad, Hanan Abdulrahman Alrubaia, Albandari Talq Alotaibi, Ossama Zakaria

**Affiliations:** 1 Department of Surgery, College of Medicine, King Faisal University, Al Hofuf, Saudi Arabia; 2 Department of Family and Community Medicine, College of Medicine, King Faisal University, Al Hofuf, Saudi Arabia; 3 Department of Pediatric Surgery, Maternity and Children’s Hospital, Al-Ahsa, Saudi Arabia; 4 College of Medicine, King Faisal University, Al Hofuf, Saudi Arabia

**Keywords:** perceptions, nocturnal enuresis, children

## Abstract

Nocturnal enuresis (NE) has been associated with neurodevelopmental disorders such as autism spectrum disorder, attention deficit or hyperactivity disorder, and intellectual disability. This study aimed to assess parents’ perception of NE in children in the eastern region of Saudi Arabia. We conducted a cross-sectional study from May to August 2023, including parents aged ≥18 years living in the area. We administered an online questionnaire to assess parents’ knowledge and attitudes toward NE and its treatment. A total of 616 parents completed the questionnaire, 71.4% of which were women, 35% were aged between 25 and 35 years, 75% were married, 65% had a university degree, and 49% had three or more children. In total, 70% demonstrated a good overall knowledge about NE and its treatment, and nearly 60% had a positive attitude toward the condition. Univariate and multivariate ordinal logistic regression analyses revealed that female sex, a higher level of education, and having more than one child were associated with a higher score regarding attitude toward treatment. The level of education and the number of children were predictors of knowledge and a positive attitude toward NE in children.

## INTRODUCTION

The International Children’s Continence Society defines nocturnal enuresis (NE) as involuntary urine loss during sleep in children aged ≥5 years [1,2]. NE is commonly known as ‘bed-wetting’ and affects boys more frequently than girls [3]. The prevalence of NE changes with age, affecting 10–15% of children below the age of 6, 5% of children below the age of 10, and 0.5–1% of teenagers and young adults [4].

NE is classified as monosymptomatic, with no other lower urinary tract symptoms, or non-monosymptomatic, when it is associated with other lower urinary tract symptoms [2]. These additional symptoms indicate a lower urinary tract dysfunction and include pain, increased or decreased frequency of urination, urgency, hesitancy, straining, weak or intermittent stream, and daytime incontinence. Monosymptomatic NE is further classified into primary and secondary NE [2,3]. Primary NE occurs in children who have not yet achieved consistent bladder control, and secondary NE occurs in children who have previously maintained bladder control for at least 6 months [1,5]. Bladder control is achieved when there is no spontaneous urine leakage. Nighttime bladder control is not typically achieved until 5–7 years of age, but it is accomplished months or years after daytime bladder control [6]. NE occurs when the child cannot wake up to urinate because of excess urine in their bladder [7]. This may be caused by several linked factors, including delayed maturation, genetics, nocturnal polyuria, sleep disturbances, small bladder capacity, and detrusor overactivity [8,9].

NE has been associated with neurodevelopmental disorders such as autism spectrum disorder, attention deficit or hyperactivity disorder, and intellectual disability [10–12]. Other factors that have been linked to this condition include lack of breastfeeding, poor academic performance, lower level of father’s education, and pinworm infestations [13].

Several studies have evaluated the prevalence of NE in Saudi Arabia and have noted variations across different regions [13–17]. A recent study assessed the prevalence of NE in several cities in Saudi Arabia and found the prevalence to be 31.2% [18].

Parents and society typically expect children to develop urinary continence by the age of 6 years. As children get older, this expectation increases. If they do not achieve bladder control, they may face stigmatization, resulting in low self-esteem and psychological issues [19,20]. The likelihood of parents taking their child to a physician depends on their perception of NE. In addition, parents and physicians have differing views on NE in children [21]. The parents’ perceptions have an important role in establishing the diagnosis and initiating treatment. However, overwhelming concern from parents or society may impose psychological effects on children. Therefore, determining parents’ knowledge and attitude toward NE is crucial to outline factors that influence its identification. Given that limited research is available about parents’ knowledge of NE in the eastern province of Saudi Arabia, this study aimed to assess the perception of NE in a group of parents from this region.

## METHODS

We carried out a cross-sectional study from May to August 2023 in the eastern province of Saudi Arabia. We included parents aged ≥18 years living in the area; individuals under 18 years of age or living outside the province were excluded. With a predetermined level of tolerable error of 5% and an assumption that 50% of the population possesses knowledge about NE, the sample size was calculated to be 385, while maintaining a type I error rate of 5% (α = 0.05). After adjustments for a response rate of 80%, the sample size was calculated to be 481.

We used non-probability convenience sampling to recruit parents who met the inclusion criteria. We distributed an online questionnaire created in Google Forms through social media channels to parents selected via simple random sampling. The questionnaire was written in Arabic and divided into four sections. The first section focused on demographics (age, sex, marital status, educational level, and number of children). The second section included three questions designed to assess the depth of knowledge about NE; response choices were ‘yes’ (3 points), ‘I don’t know’ (2 points), and ‘no’ (1 point), with a maximum possible score of 9 points. The third section included four questions designed to assess knowledge about the treatment of NE; response choices were ‘yes’ (3 points), ‘I don’t know’ (2 points), and ‘no’ (1 point), with a maximum possible score of 12 points. The fourth and fifth sections included questions intended to assess attitudes toward NE and its treatment, respectively, with responses scored on a 5-point Likert scale, from ‘strongly agree’ to ‘strongly disagree’ and a maximum possible score of 20 points. A higher score was considered to indicate a higher level of knowledge and a positive attitude. Bloom’s cut-off points were used to determine the overall level of knowledge and attitude (outcome measures), as follows:

1. Knowledge about NE: a score of ≥80% (7 points) was deemed to indicate good knowledge, ≥60% (5 points) intermediate knowledge, and <60% poor knowledge.
2. Knowledge about the treatment of NE: a score of ≥80% (9 points) was deemed to indicate good knowledge, ≥60% (7 points) intermediate knowledge, and <60% poor knowledge.
3. Attitudes toward NE and its treatment: a score of ≥80% (16 points) was deemed to indicate a positive attitude, 60–80% (12 points) a neutral attitude, and <60% a negative attitude.

### Questionnaire validation

The questionnaire was designed in Arabic language and its linguistic clarity was verified by language experts. The LAWSHE method was employed to assess the content validity of the questionnaire. Five experts were consulted to provide their opinions on each item in the questionnaire, and the content validity ratio was calculated accordingly. Questions with a content validity ratio below 0.99 were eliminated from the questionnaire. The construct validity and reliability of the questionnaire were assessed in a pilot study involving 70 participants. However, the data collected from these participants were not included in the final analysis and dissemination of the questionnaire results.

The study used the partial least squares structural equation modelling (PLS-SEM) approach in SmartPLS v.4 (SmartPLS). The main objective was to determine the factor loadings for each domain of the questionnaire to evaluate the validity (convergent and discriminant) and reliability (internal consistency) of four domains developed based on data from previous research on NE [18–21]. The four domains were the following: 1) knowledge about NE; 2) knowledge about the treatment of NE; 3) attitudes toward NE; 4) attitudes toward the treatment of NE. Questions with a factor loading below 0.4 were excluded from the questionnaire. Specifically, we removed one question from the domain of knowledge about NE, one question from the domain of knowledge about the treatment of NE, and one question from the domain of attitudes toward the treatment of NE.

**Figure 1:**
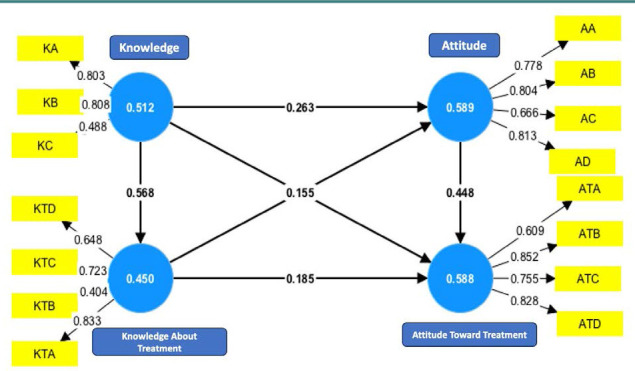
Measurement model and factor loadings for each questionnaire construct. Yellow rectangles represent the questions (factors and items), and blue circles represent the constructs (latent variables and domains). Large arrows between circles represent the relationship between latent variables, whereas small arrows between rectangles and circles represent factor loadings in latent variables.

The composite reliability (rho_c) of all constructs exceeded the threshold of 0.7 when assessed using the measurement model presented in Figure 1, suggesting that the questionnaire had satisfactory internal consistency. The average variance extracted values of all constructs exceeded or were close to 0.5, indicating the presence of convergent validity (Supplementary Table 1). The Fornell and Larker criteria were used to assess discriminant validity (Supplementary Table 2). The application of consistent PLS-SEM bootstrapping to assess the approximate model fit resulted in a standardized root mean square residual value of 0.059 (95% confidence interval (CI) 0.054–0.062), below the threshold of 0.1, suggesting that the model exhibited an acceptable fit.

**Table 1:** Demographic characteristics of the study population (*n* = 616)

Variable	Frequency (%)
**Sex**	
Male	174 (28.2%)
Female	442 (71.8%)
**Age**	
<25 years	131 (21.3%)
25–35 years	164 (26.6%)
36–50 years	218 (35.4%)
>50 years	103 (16.7%)
**Marital status**	
Single	152 (24.7%)
Married	464 (75.3%)
**Level of education**	
Uneducated	13 (2.1%)
Less than a bachelor’s degree	132 (21.4%)
Bachelor’s degree	401 (65.1%)
Postgraduate degree	70 (11.4%)
**Number of children**	
No children	164 (26.6%)
One child	78 (12.7%)
Two children	70 (11.4%)
Three or more children	304 (49.4%)

Supplementary Material

### Statistical analysis

Data were analyzed using IBM SPSS version 25 (IBM Corp.). Categorical variables are reported as frequencies. We performed univariate and multivariate ordinal logistic regression analyses to explore associations between demographic variables and the levels of knowledge and attitudes, and to assess the effect of knowledge on attitudes. The results are reported as crude odds ratios (CORs) for the univariate analysis and adjusted odds ratios (AORs) for the multivariate analysis, along with their 95% CIs. *P* values of <0.05 were considered statistically significant.

## MATERIAL AND METHODS

### Demographics

A total of 616 parents completed the questionnaire, 71.4% of which were women, 35% were aged between 25 and 35 years, 75% were married, 65% had a university degree, and 49% had three or more children (Table 1).

### Knowledge about NE and its treatment

**Figure 2:**
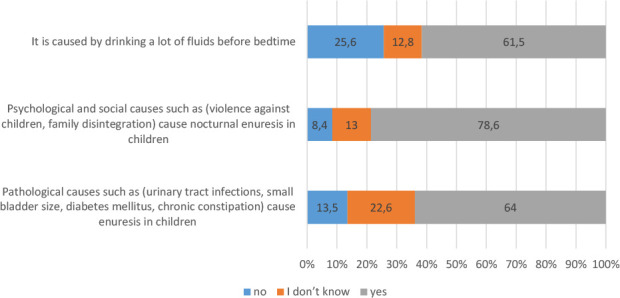
The distribution of answers to questions related to knowledge about NE (*n* = 616)

**Figure 3:**
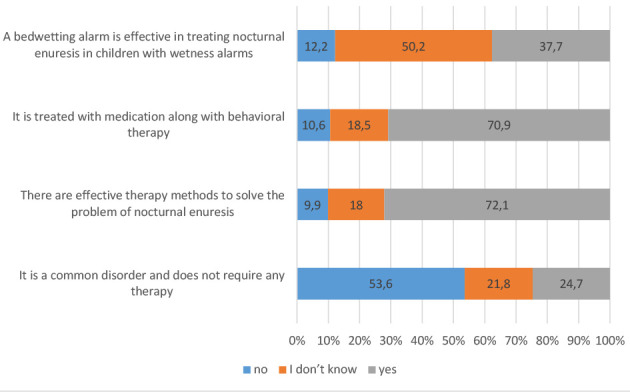
The distribution of answers to questions related to knowledge about the treatment of NE (*n* = 616)

In total, 78% of the participants were aware that psychological and social factors (e.g., violence against children or family disintegration) can cause NE in children, and 64% were aware of the pathological causes of NE in children (e.g., urinary tract infections, small bladder size, diabetes mellitus, chronic constipation) (Figure 2). In addition, 72% were aware that there are effective treatments for NE, and 70% were aware that NE can be treated with medication along with behavioral therapy (Figure 3). Approximately 70% and 67% of the participants demonstrated a good overall knowledge regarding the disease and its treatment, respectively (Table 2). The univariate ordinal logistic regression analysis indicated that having more than three children, being a woman, being married, and having a higher level of education were significantly associated with a higher knowledge score. By contrast, the multivariate analysis indicated only having a higher level of education and having more than three children as significant predictors of good knowledge about NE (Table 3).

**Table 2:** Distribution of knowledge and attitude scores (*n* = 616)

Variable	Frequency (%)
**Knowledge about NE**	
Poor	19 (3.1%)
Moderate	155 (25.2%)
Good	442 (71.8%)
**Knowledge about treatment of NE**	
Poor	5 (0.8%)
Moderate	198 (32.1%)
Good	413 (67.0%)
**Attitude toward NE**	
Negative	43 (7.0 %)
Neutral	210 (34.1%)
Positive	363 (58.9%)
**Attitude toward treatment of NE**	
Negative	28 (4.5%)
Neutral	217 (35.2%)
Positive	371 (60.2%)

**Table 3:** Association between demographic variables and knowledge using univariate and multivariate ordinal logistic regression

Predictors	AOR (95% CI)	*P* value	COR (95% CI)	*P* value
**Sex**				
Female / male	1.20 (0.87–1.65)	0.262	1.40 (1.02–1.90)	0.035*
**Age**				
25–35 years / <25 years	0.72 (0.45–1.15)	0.168	0.97 (0.65–1.44)	0.871
36–50 years / <25 years	0.46 (0.27–0.77)	0.003*	1.09 (0.75–1.59)	0.635
Over >50 years / <25 years	0.56 (0.30–1.03)	0.063	1.43 (0.92–2.24)	0.114
**Marital status**				
Married / single	1.04 (0.62–1.75)	0.871	1.38 (1.00–1.89)	0.048*
**Education level**				
Less than a bachelor’s degree / uneducated	5.18 (1.94–13.86)	<–0.001*	6.40 (2.43–16.90)	<0.001*
Bachelor’s degree / uneducated	8.01 (3.09–20.84)	<–0.001*	9.79 (3.83–25.10)	<0.001*
Postgraduate degree / uneducated	7.92 (2.83–22.24)	<–0.001*	9.71 (3.55–26.70)	<0.001*
**Number of children**				
One child / no child	0.58 (0.31–1.06)	0.076	0.53 (0.32–1.87)	0.263
Two children / no child	1.41 (0.73–2.72)	0.302	1.20 (0.73–1.99)	0.469
Three or more children / no child	2.60 (1.43–4.76)	0.002*	1.77 (1.28–2.47)	<0.001*

* Significant

The univariate analysis indicated that being a woman, having an age below 25 years, having more than three children, and possessing a higher level of education were associated with a higher level of awareness about the treatment of NE. The multivariate analysis revealed similar findings, with the exception of the number of children (Table 4).

**Table 4:** Association between demographic variables and knowledge about the treatment of NE using univariate and multivariate ordinal logistic regression

Predictors	AOR (95% CI)	*P* value	COR (95% CI)	*P* value
**Sex**				
Female / male	1.61 (1.17–2.22)	0.003*	1.82 (1.33–2.5)	<0.001*
**Age**				
25–35 years / <25 years	0.60 (0.37–0.97)	0.038*	0.63 (0.41–0.95)	0.028*
36–50 years / <25 years	0.50 (0.30–0.85)	0.01*	0.76 (0.52–1.11)	0.157
Over >50 years / <25 years	0.39 (0.21–0.71)	0.002*	0.60 (0.38–0.95)	0.031*
**Marital status**			0.74 (0.53–1.02)	
Married / single	0.69 (0.41–1.16)	0.161		0.069
**Education level**			3.41 (1.27–9.03)	
Less than a bachelor’s degree / uneducated	2.81 (1.05–7.48)	0.038*	6.03 (2.31–15.55)	0.014*
Bachelor’s degree / uneducated	4.90 (1.89–12.72)	<0.001*	8.39 (2.98–23.39)	<0.001*
Postgraduate degree / uneducated	8.48 (2.99–24.03)	<0.001*	0.68 (0.23–1.63)	<0.001*
**Number of children**				
One child / no child	0.63 (0.35–1.15)	0.132	0.68 (0.23–1.63)	0.301
Two children / no child	1.29 (0.67–2.47)	0.45	0.81 (0.49–1.32)	0.389
Three or more children / no child	0.91 (0.65–1.28)	0.585	1.83 (1.01–3.32)	0.047*

* Significant

### Attitudes toward enuresis and its treatment

**Figure 4:**
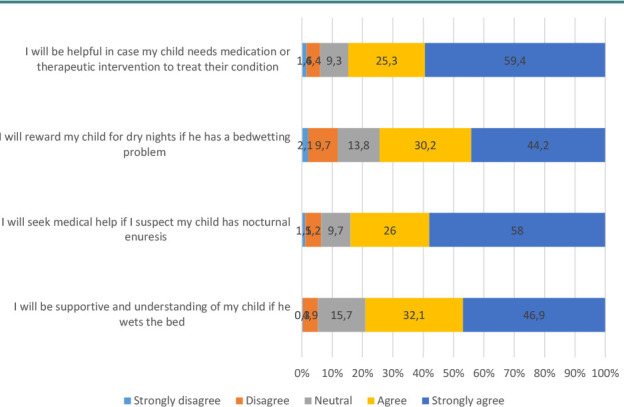
The distribution of answers to questions related to attitude toward NE (*n* = 616)

**Figure 5:**
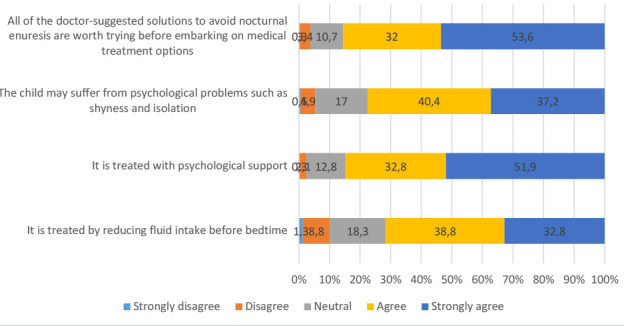
The distribution of answers to questions related to attitude toward the treatment of NE (*n* = 616)

In total, 59% of the participants strongly agreed that they would be helpful in case their children needed medication or therapeutic intervention to treat NE, and 46% strongly agreed that they would be supportive in case their children wet the bed (Figure 4). Additionally, 50% percent strongly agreed that all doctor-suggested solutions to avoid NE are worth trying before embarking on medical treatment options (Figure 5). Almost 60% of the participants had an overall positive attitude toward NE and its treatment (Table 2).

Both univariate and multivariate analyses indicated a positive association of a higher level of education and a higher number of children with a higher score regarding attitude toward NE (Table 5). Conversely, the univariate analysis demonstrated that being a woman, possessing a higher level of education, and having more than one child were significantly associated with a higher score regarding attitude toward treatment (Table 6).

**Table 5:** Association between demographic variables and attitudes toward NE using univariate and multivariate ordinal logistic regression

Predictors	AOR (95% CI)	*P* value	COR (95% CI)	*P* value
**Sex**				
Female / male	1.22 (0.89–1.68)	0.218	1.34 (0.98–1.83)	0.064
**Age**				
25–35 years / <25 years	0.91 (0.56–1.46)	0.687	0.97 (0.64–1.46)	0.871
36–50 years / <25 years	0.61 (0.37–1.02)	0.06*	0.87 (0.59–1.28)	0.478
Over >50 years / <25 years	0.61 (0.33–1.11)	0.103	0.93 (0.59–1.45)	0.739
**Marital status**				
Married / single	1.55 (0.89–2.69)	0.12	1.00 (0.72–1.38)	0.993
**Education level**				
Less than a bachelor’s degree / uneducated	2.76 (1.05–7.28)	0.039*	2.92 (1.13–7.50)	0.025*
Bachelor’s degree / uneducated	4.45 (1.75–11.46)	0.002	4.87 (1.95–12.18)	<0.001*
Postgraduate degree / uneducated	6.35 (2.30–17.65)	<0.001*	6.29 (2.33–16.90)	<0.001*
**Number of children**				
One child / no child	2.41 (1.41–4.14)	0.001*	2.62 (1.60–4.30)	<0.001*
Two children / no child	1.87 (1.00–3.52)	0.051	2.11 (1.19–3.76)	0.011*
Three or more children / no child	2.46 (1.50–4.02)	<0.001*	2.38 (1.52–3.76)	<0.001*

* Significant

**Table 6:** Association between demographic variables and attitudes toward the treatment of NE using univariate and multivariate ordinal logistic regression

Predictors	AOR (95% CI)	*P* value	COR (95% CI)	*P* value
**Sex**				
Female / male	1.36 (0.93–1.97)	0.107	1.63 (1.15–2.31)	0.006*
**Age**				
25–35 years / <25 years	0.76 (0.44–1.30)	0.312	0.91 (0.58–1.44)	0.692
36–50 years / <25 years	0.73 (0.41–1.34)	0.314	1.32 (0.85–2.05)	0.212
Over >50 years / <25 years	0.76 (0.37–1.53)	0.433	1.41 (0.84–2.40)	0.198
**Marital status**				
Married / single	1.36 (0.75–2.48)	0.32	1.42 (0.98–2.03)	0.061
**Education level**				
Less than a bachelor’s degree / uneducated	2.38 (0.74–7.73)	0.145	2.88 (0.92–9.16)	0.07
Bachelor’s degree / uneducated	6.42 (2.05–20.36)	0.001*	7.84 (2.57–24.40)	<.001*
Postgraduate degree / uneducated	6.68 (1.94–23.37)	0.003*	7.94 (2.38–27.00)	<.001*
**Number of children**				
One child / no child	1.86 (0.95–3.69)	0.073	1.73 (1.02–2.95)	0.044*
Two children / no child	1.26 (0.65–2.43)	0.491	1.46 (0.78–2.75)	0.242
Three or more children / no child	2.76 (1.55–4.93)	<.001*	2.88 (1.75–4.75)	<.001*

* Significant

### Association between knowledge and attitude scores

The univariate ordinal regression analysis revealed that a higher score regarding knowledge about NE and its treatment and a higher score regarding attitude toward NE were associated with a more positive attitude toward treatment. In the multivariate analysis, all aforementioned predictors, except for the score regarding knowledge about treatment, were associated with a higher score regarding attitude toward treatment (Table 7).

**Table 7:** The effects of knowledge about enuresis, knowledge about enuresis treatment and attitude toward enuresis scores on attitude toward enuresis treatment scores using univariate and multivariate ordinal logistic regression

	Univariate analysis		Multivariate analysis	
**Predictors**	**COR (95% CI)**	***P* value**	**AOR (95% CI)**	***P* value**
**Knowledge about NE**				
Moderate / low	1.98 (0.72–5.57)	0.189	5.71 (2.23–14.80)	<0.001*
Good / low	3.67 (1.31–10.42)	0.014*	17.65 (7.02–45.00)	<0.001*
**Knowledge about the treatment of NE**				
Moderate / low	0.83 (0.14–4.92)	0.833	1.27 (0.24–6.81)	0.778
Good / low	2.04 (0.35–12.24)	0.419	6.38 (1.24–34.27)	0.027*
**Attitude toward NE**				
Neutral / negative	6.44 (2.97–14.44)	<0.001*	11.70 (5.59–25.50)	<0.001*
Positive / negative	17.70 (7.88–41.01)	<0.001*	44.30 (20.98–97.50)	<0.001*

* Significant

## DISCUSSION

The most obvious finding to emerge from the analysis is that 78% of the participants were aware of the psychological and social causes of NE, and 64% were aware of the pathological causes of NE in children, indicating a commendable level of understanding about the multifaceted causes of the condition. The awareness of psychological and social causes noted in the present study is in line with the findings of Bulut *et al*. regarding the growing recognition of psychosocial factors of NE [22]. Further, the awareness of pathological causes in the present study is consistent with the results of Schultz Lampel *et al*., who noted similar awareness levels among the participants of their study [23].

Approximately 72% of the participants were aware about the availability of effective treatments for NE. This finding indicates that a significant proportion of parents recognize that solutions exist for managing NE [24]. Further, 70% of the participants were aware that NE can be treated with a combination of medication and behavioral therapy, highlighting positive awareness about the holistic approach required to manage the condition effectively [25]. Based on the overall knowledge score, the majority of the participants possessed a satisfactory understanding of both the condition and its treatment options.

In the current study, several demographic factors were found to be associated with increased knowledge scores, including having more than three children, being a woman, being married, and having a higher level of education. In the multivariate analysis, only having a higher educational level and having more than three children remained as significant predictors of a higher knowledge score. This finding suggests that education and family size are the primary factors influencing parents’ knowledge about NE and its treatment [26].

The univariate analysis indicated that female sex, age below 25 years, having more than three children, and a higher level of education were associated with increased awareness about the treatment of NE. The multivariate analysis confirmed these associations, except for the number of children. These findings imply that sex, age, and level of education consistently play a role in shaping parents’ knowledge about the treatment of NE [26].

Approximately 59% of the participants strongly agreed that they would be helpful if their children required medication or therapeutic intervention for NE. This finding aligns with the study of Schlomer *et al*., wherein participants exhibited similar levels of positive attitude toward the health issues of their child [27]. Such data demonstrate a positive and supportive stance among a considerable proportion of parents. Additionally, 46% of the participants in the present study strongly agreed that they would be supportive if their children experienced bed-wetting, indicating a compassionate and understanding attitude toward a common issue that many children face.

Half of the participants strongly agreed that they would try all doctor-suggested solutions before considering medical treatment options for NE. This finding reflects a preference for exploring non-invasive approaches before resorting to more intensive medical interventions [28]. Nearly 60% of the participants displayed an overall positive attitude toward NE and its treatment. This is a promising finding, as it indicates that the majority of parents are receptive and optimistic about addressing the condition [29].

Both univariate and multivariate analyses indicated that a higher educational level and an increased number of children were positively associated with a higher attitude score. This finding suggests that individuals with a higher educational level and those with more children tend to demonstrate more positive attitudes toward NE and its treatment [15]. The univariate analysis revealed that being a woman, possessing a higher level of education, and having more than one child were associated with a higher attitude score, specifically toward treatment. The multivariate analysis reinforced these associations, indicating that sex, educational level, and family size consistently have a role in shaping parents’ attitudes toward treatment.

The univariate ordinal regression analysis showed that higher scores on knowledge about NE and its treatment and higher scores on attitude toward NE were linked to more positive attitudes toward treatment. This finding suggests that parents who possess better understanding and more positive attitudes are more likely to approach treatment options with optimism and receptiveness. The positive association noted between knowledge and attitudes toward treatment in the present study aligns with the conclusions drawn in the study conducted by Yilmaz *et al*. [30]. In the multivariate analysis, after accounting for multiple predictors, we found that all factors remained associated with higher scores on attitude toward treatment. However, an interesting finding emerged: the score regarding knowledge about treatment did not significantly predict the score regarding attitude toward treatment.

Despite the valuable insights drawn from this study, there are several limitations that need to be acknowledged. First, the study’s cross-sectional design has captured only an overview of the participants’ knowledge and attitudes at a specific timepoint, preventing the exploration of potential changes over a longer period. Second, the reliance on self-reported data might have introduced recall bias or social desirability bias, thereby affecting the accuracy of the responses. Third, the study sample predominantly consisted of women, potentially limiting the generalizability of the findings to a broader population. The study’s focus on specific demographic factors might have overlooked other variables that could affect knowledge and attitudes toward NE.

## CONCLUSION

The majority of the participants demonstrated an awareness of the causative factors and treatment methods for NE. Positive attitudes toward treatment strategies were also prevalent. The level of education and the number of children emerged as significant predictors of both knowledge and attitudes toward NE and its treatment. These findings can inform targeted educational interventions and healthcare strategies to enhance the understanding and foster positive attitudes toward the management of NE.

## Data Availability

The data and materials are available on reasonable request from the corresponding author.
