# Supplementary material for: Parental perception of nocturnal enuresis in a local region of Saudi Arabia
Source: J Med Life. 2024 Jan;17(1):73–80. doi: 10.25122/jml-2023-0423 (PMC11080501; doi:10.25122/jml-2023-0423)
Supplement: Supplementary file 1 [file JMedLife-17-073-s001.pdf]

Supplementary file. The results of validity and reliability testing

Supplementary Table 1. Composite reliability and average variance extracted values for each construct to determine internal consistency and convergence validity

Construct (domain)	Composite reliability (rho_c)	Average variance extracted
Attitude toward NE	0.85	0.59
Attitude toward treatment of NE	0.85	0.59
Knowledge about treatment of NE	0.76	0.47
Knowledge about NE	0.75	0.51

Supplementary Table 2. The correlation matrix used to assess divergent validity among the various constructs, based on Fornell and Larker criteria

Construct (domain)	Attitude toward NE	Attitude toward treatment of NE	Knowledge about treatment of NE	Knowledge about NE
Attitude toward NE	0.77			
Attitude toward treatment of NE	0.61	0.77		
Knowledge about treatment of NE	0.50	0.50	0.67	
Knowledge about NE	0.46	0.47	0.57	0.71

© 2024 by the authors. This article is an open access article distributed under the terms and conditions of the Creative Commons Attribution (CC BY 4.0) license.
